# Effectiveness, safety/tolerability of OBV/PTV/r ± DSV in patients with HCV genotype 1 or 4 with/without HIV-1 co-infection, chronic kidney disease (CKD) stage IIIb-V and dialysis in Spanish clinical practice – Vie-KinD study

**DOI:** 10.1371/journal.pone.0221567

**Published:** 2019-09-24

**Authors:** María-Carlota Londoño, Mar Riveiro-Barciela, Adriana Ahumada, Raquel Muñoz-Gómez, Mercé Roget, María J. Devesa-Medina, Miguel Ángel Serra, Carmen A. Navascués, Carme Baliellas, Teresa Aldamiz-Echevarría, María L. Gutiérrez, Benjamín Polo-Lorduy, Isabel Carmona, Salvador Benlloch, Lucía Bonet, Javier García-Samaniego, Miguel Jiménez-Pérez, Senador Morán-Sánchez, Ángeles Castro, Manuel Delgado, Francisco Gea-Rodríguez, Ignacio Martín-Granizo, María Luisa Montes, Luís Morano, Manuel A. Castaño, Ignacio de los Santos, Montserrat Laguno, Juan Emilio Losa, Marta Montero-Alonso, Antonio Rivero, Cristina de Álvaro, Amanda Manzanares, Josep Mallolas, Guillermina Barril, Emilio González-Parra, Luisa García-Buey

**Affiliations:** 1 Liver Unit, Hospital Clínic/IDIBAPS, Barcelona, Barcelona, Spain; 2 Centro de Investigación Biomédica en Red de Enfermedades Hepáticas y Digestivas (CIBERhed), Instituto de Salud Carlos III, Madrid, Madrid, Spain; 3 Liver Unit, Internal Medicine Department, Hospital Vall d'Hebron, Barcelona, Barcelona, Spain; 4 Department of Gastroenterology, Hospital General Universitario Gregorio Marañón, Madrid, Madrid, Spain; 5 Department of Gastroenterology, Hospital General Universitario 12 de Octubre, Madrid, Madrid, Spain; 6 Liver Unit, Consorci Sanitari de Terrassa, Terrassa, Barcelona, Spain; 7 Department of Gastroenterology, Hospital Universitario Clínico San Carlos, Madrid, Madrid, Spain; 8 Digestive Medicine Service, Hospital Clínico Universitario de Valencia, Universidad de Valencia, Valencia, Spain; 9 Department of Gastroenterology, Hospital Universitario Central de Asturias, Oviedo, Asturias, Spain; 10 Liver Unit, Hospital de Bellvitge, Institut d'Investigació Biomèdica de Bellvitge, University of Barcelona, L'Hospitalet de Llobregat, Barcelona, Spain; 11 Infectious Diseases-HIV Hospital General Universitario Gregorio Marañón (IiSGM), Madrid, Madrid, Spain; 12 Department of Gastroenterology, Hospital Universitario Fundación Alcorcón, Alcorcón, Madrid, Spain; 13 Digestive Diseases Unit, Hospital Universitario Fundación Jiménez Díaz, Madrid, Madrid, Spain; 14 Digestive Disease Unit, Hospital Universitario Virgen Macarena, Sevilla, Sevilla, Spain; 15 Department of Hepatology, Hospital Universitario y Politécnico La Fe, Valencia, Valencia, Spain; 16 Department of Gastroenterology, Hospital Universitario Son Espases, Palma de Mallorca, Mallorca, Spain; 17 Liver Unit, Hospital Universitario La Paz/IdiPaz, Madrid, Madrid, Spain; 18 Hospital Regional Universitario de Málaga, Málaga, Spain; 19 Hospital General Universitario Santa Lucía, Cartagena, Murcia, Spain; 20 Complejo Hospitalario Universitario A Coruña, A Coruña, Spain; 21 Department of Gastroenterology, Hospital Universitario Ramón y Cajal, Madrid, Madrid, Spain; 22 Department of Gastroenterology, Hospital Universitario Álvaro Cunqueiro, Vigo, Pontevedra, Spain; 23 HIV Unit, Hospital Universitario La Paz/IdiPaz, Madrid, Madrid, Spain; 24 Infectious Disease Unit, Internal Medicine Department, Hospital Universitario Álvaro Cunqueiro, Vigo, Pontevedra, Spain; 25 Department of Internal Medicine, Hospital Universitario La Princesa, Madrid, Madrid, Spain; 26 HIV Unit, Infectious Diseases Service, Hospital Clínic/IDIBAPS, Barcelona, Barcelona, Spain; 27 Infectious Diseases Unit, Hospital Universitario Fundación Alcorcón, Alcorcón, Madrid, Spain; 28 Infectious Diseases Unit, Hospital Universitario y Politécnico La Fe, Valencia, Valencia, Spain; 29 Infectious Diseases Unit, Hospital Universitario Reina Sofía de Córdoba, Instituto Maimónides de Investigación Biomédica de Córdoba (IMIBIC), Universidad de Córdoba, Córdoba, Spain; 30 Medical Department & Quality Assurance, ABBVIE, Madrid, Madrid, Spain; 31 Nephrology Unit, Hospital Universitario La Princesa, Madrid, Madrid, Spain; 32 Nephrology Unit, Hospital Universitario Fundación Jiménez Díaz, Madrid, Madrid, Spain; 33 Liver Unit, Hospital Universitario La Princesa, Madrid, Madrid, Spain; Centers for Disease Control and Prevention, UNITED STATES

## Abstract

**Background and aims:**

Limited data are available on the effectiveness and tolerability of direct-acting antivirals (DAAs) therapies in the real world for HCV-infected patients with comorbidities. This study aimed to describe the effectiveness of OBV/PTV/r ± DSV (3D/2D regimen) with or without ribavirin (RBV) in HCV or HCV/HIV co-infected patients with GT1/GT4 and CKD (IIIb-V stages), including those under hemodialysis and peritoneal dialysis in routine clinical practice in Spain in 2015.

**Material and methods:**

Non-interventional, retrospective, multicenter data collection study in 31 Spanish sites. Socio-demographic, clinical variables, study treatment characteristics, effectiveness and tolerability data were collected from medical records.

**Results:**

Data from 135 patients with a mean age (SD) of 58.3 (11.4) years were analyzed: 92.6% GT1 (81.6% GT1b and 17.6% GT1a) and 7.4% GT4, 14 (10.4%) HIV/HCV co-infected, 19.0% with fibrosis F3 and 28.1% F4 by FibroScan®, 52.6% were previously treated with pegIFN and RBV. 11.1%, 14.8% and 74.1% of patients had CKD stage IIIb, IV and V respectively. 68.9% of patients were on hemodialysis; 8.9% on peritoneal dialysis and 38.5% had history of renal transplant. A total of 125 (96.2%) of 135 patients were treated with 3D, 10 (7.4%) with 2D and 30.4% received RBV. The overall intention-to-treat (ITT) sustained virologic response at week 12 (SVR12) was 92.6% (125/135) and the overall modified-ITT (mITT) SVR12 was 99.2% (125/126). The SVR12 rates (ITT) per sub-groups were: HCV mono-infected (91.7%), HCV/HIV co-infected (100%), GT1 (92.0%), GT4 (100%), CKD stage IIIb (86.7%), stage IV (95%) and stage V (93%). Among the 10 non-SVR there was only 1 virologic failure (0.7%); 4 patients had missing data due lost to follow up (3.0%) and 5 patients discontinued 3D/2D regimen (3.7%): 4 due to severe adverse events (including 3 deaths) and 1 patient´s decision.

**Conclusions:**

These results have shown that 3D/2D regimens are effective and tolerable in patients with advanced CKD including those in dialysis with GT 1 or 4 chronic HCV mono-infection and HIV/HCV coinfection in a real-life cohort. The overall SVR12 rates were 92.6% (ITT) and 99.2% (mITT) without clinically relevant changes in eGFR until 12 weeks post-treatment. These results are consistent with those reported in clinical trials.

## Introduction

Hepatitis C virus (HCV) infection is a major health problem affecting more than 71 million people worldwide [1]. In Spain, the latest studies show an HCV infection prevalence in the general population of between 0.87% and 1.19% [2,3]. The serious clinical outcomes of chronic infection are cirrhosis, portal hypertension, hepatic decompensation and the development of hepatocellular carcinoma, which leads to liver-related mortality. Approximately 10–15% of patients with chronic HCV infection will progress to cirrhosis and up to 30% of them will progress to hepatic decompensation [4], with a significant impact on health-related quality of life [5,6].

Chronic kidney disease (CKD) is a major health problem in developed societies, affecting 11% of the population, and it is an underdiagnosed disease [7]. In clinical practice, Park et al. reported that HCV patients have a 23% greater risk of having or developing CKD compared to the general population [8]. The estimated prevalence of HCV infection in patients on haemodialysis is around 5.6%, in Spain, with wide variations both geographically and between units in the same country [9]. Moreover, Fabrizi et al. reported that anti-HCV positive patients on dialysis have an increased risk of cardiovascular mortality (adjusted RR 1.26; 95% CI, 1.10; 1.45) or liver-related mortality (adjusted RR 3.82; 95% CI, 1.92; 7.61) compared with anti-HCV negative patients [10]. In addition, HCV infection adversely decreases the health-related quality of life of these patients [11].

HIV and HCV viruses share some routes of transmission (i.e. intravenous drug users [12,13]) and consequently co-infection is common. About 25% of HIV-infected individuals worldwide are also co-infected with HCV [14]. Co-infected patients progress faster to cirrhosis and end-stage liver disease and are more likely to have HIV-related kidney disease than patients with HCV alone [15].

The introduction of direct-acting antivirals (DAAs) in 2011[16], and more recently the IFN-free DAA regimens, have increased the number of patients who respond to treatment, and marked a new era of HCV therapy [17]. Moreover, the improvement of quality of life, efficacy and overall survival of patients with chronic HCV infection has constituted an enormous medical advance, since these new agents can cure HCV infection. Therefore, clinical practice guidelines on hepatitis C treatment (i.e. EASL and AASLD/IDSA) recommend therapies in patients with HCV infection and CKD with severe renal impairment or end-stage renal disease (ESRD), including those on haemodialysis. Although, at the time of writing, there are DAA molecules that are safe in HCV genotype (GT) 1- or 4-infected patients with severe renal impairment, sofosbuvir is still not recommended in patients with an estimated glomerular filtration rate (eGFR) less than 30 ml/min [18,19]. Ombitasvir (OBV) / paritaprevir (PTV) / ritonavir(r) and dasabuvir (DSV) (3D) or OBV/PTV/r (2D) with or without ribavirin (RBV) regimens have shown very high efficacy and safety results in clinical trials in HCV patients with and without CKD. These molecules are metabolised in the liver and do not require dose-adjustment in CKD patients according to label information. This affirmation is based on pharmacokinetic studies comparing CKD patients (including those with ESRD) with healthy volunteers, which conclude that 3D and 2D regimens have the same pharmacokinetic profile and similar parameters in both populations [20,21].

The RUBY-I study has shown very good efficacy and safety data: SVR12 90% per intention-to-treat (ITT) population analysis and SVR12 95% per modified-ITT (mITT) analysis were observed in cohort 1 [22] that included non-cirrhotic patients with stage 4 or 5 CKD and HCV GT1 infection who had never been treated. A SVR12 rate of 96% was observed in cohort 2 of RUBY-I [23] in GT1-infected patients with compensated cirrhosis, including those previously treated with IFN/pegIFN±RBV. The regimen was generally well tolerated for these groups of patients. Moreover, results of the RUBY-II [24] study in HCV non-cirrhotic, treatment-naïve patients with stage 4 or 5 CKD, including those receiving dialysis, showed that the RBV-free regimen of both 3D and 2D resulted in an overall ITT SVR12 rate of 94% for GT1a and GT4 and a mITT SVR12 rate of 100%, with no on-treatment virological failure or relapse.

The main purpose of this study was to describe patterns of real-world effectiveness and safety/tolerability of the treatment with 3D/2D ± RBV in GT1 and GT4 HCV-infected patients with CKD stages IIIb to V, including those in dialysis.

## Material and methods

A non-interventional, retrospective, epidemiological, multicentre data collection study was designed, with the participation of 31 Spanish sites distributed across the country, which ensured a heterogeneous patient sample, reducing the likelihood of a biased study population. The study was approved by the Spanish Agency of Medicines and Medical Devices and classified as EPA-OD (code ABB-OMD-2015-01). The study was evaluated and approved by the Independent Ethics Committee of different hospitals involved, listed in S1 Text.

This study was designed, implemented and reported in accordance with the Guidelines for Good Pharmacoepidemiology Practices (GPP) of the International Society for Pharmacoepidemiology (ISPE 2007), and with the ethical principles laid down in the Declaration of Helsinki (http://www.wma.net/e/policy/b3.htm). The study guaranteed patient confidentiality and follows European and local data protection laws. No information identifying patients was captured. The Institutional Review Board (IRB) / Independent Ethics Committee (IEC) / Competent Authorities (CA) of all sites exempted the investigators from obtaining patient consent forms because the study was fully retrospective.

Data were collected between June and November 2016 from patients who were ≥18 years, with HCV GT1 or GT4 infection (HIV-1 co-infection with undetectable HIV baseline viral load was accepted), with CKD stage IIIb-V, fibrosis stage F0-F4 and who had started treatment with 3D or 2D, ± RBV, in 2015 for an intended duration of 12 or 24 weeks according the SmPC indications.

The exclusion criteria were: previous failures to any DAAs, diagnosis of hepatocellular carcinoma, decompensated cirrhosis, positive test result for hepatitis B surface antigen and liver or renal transplant under immunosuppressive therapy.

Data were recorded in four different time frames: at inclusion (Baseline), after 4 weeks of treatment, at end of treatment (EOT) and at 12 weeks after EOT to evaluate sustained virological response at week 12 (SVR12). Socio-demographic variables (age, gender, body mass index [BMI]), clinical variables (genotype, fibrosis stage and viral load), laboratory tests, characteristics of previous therapies and the study treatment, concomitant diseases and effectiveness and tolerability of 3D/2D ± RBV data were collected from medical records.

To assess the effectiveness of 3D/2D ± RBV treatment in the selected population, SVR12 was defined as the percentage of patients with undetectable HCV RNA 12 weeks after receiving the treatment. For this study, HCV-RNA was quantified with a limit of detection (LoD) specific to each participant site. The percentage of patients achieving SVR12 and the corresponding exact two-sided 95% confidence interval (CI) were computed based on the normal approximation method.

The primary endpoint, SVR12, was analysed in all patients who fulfilled the inclusion criteria and were treated with at least 1 dose of the 3D or 2D ± RBV treatment as ITT. The mITT population included those patients in the ITT population minus those patients for whom SVR12 assessment data were missing (unknown) and those patients who discontinued treatment.

SVR12 was also analysed by sub-groups: CKD stage (IIIb, IV and V), HCV mono-infection or HIV/HCV coinfection, genotype (GT1 or GT4) and GT1 subtype, concomitant use of RBV and renal transplant. The SVR12 bootstrap distribution of a population parameter was used to produce a bootstrapped confidence interval for the parameter’s true value. Five thousand random samples of 135 sizes were obtained to calculate SVR12 estimation and the non-parametric 95% CI. Estimated glomerular filtration rate (eGFR) was calculated using the serum creatinine value, based on the CKD-EPI creatinine equation [25]:

$$eGFR=141*\min{\left(\frac{\mathrm{Creat}}{k},\;1\right)}^{\alpha }*\max{\left(\frac{\mathrm{Creat}}{k},1\right)}^{-1.209}*0.993^{Age}*1.018\left[if\;female\right]*1.159\left[if\;black\right]$$

where: Creat is serum creatinine in μmol/l, κ is 61.9 for females and 79.6 for males, α is -0.329 for females and -0.411 for males, min indicates the minimum of Creat /κ or 1, and max indicates the maximum of Creat /κ or 1.

Stage IIIb, IV and V were defined (according to the KDIGO guidelines) [26] as an eGFR: 30–44 ml/min/1.73m^2^; 15–29 ml/min/1.73m^2^ and eGFR<15 ml/min/1.73m^2^ or dialysis, respectively.

### Statistical analysis

Qualitative variables were summarised in a table including absolute and relative frequencies per treatment group and in the whole population. Quantitative variables were provided using valid n, mean, standard deviation, two-sided 95% confidence interval (in primary endpoints), median, interquartile range, minimum and maximum.

Quantitative variables were compared between groups using an analysis of variance (ANOVA). Quantitative parameters without a normal distribution and ordinal parameters were compared using a non-parametric Kruskal-Wallis test. Qualitative parameters were analysed using a χ^2^ test or, if assumptions were not met, a Fisher’s exact test. The significance level was generally established at a value of α = 0.05.

Logistic regression was used to investigate the impact of the following explanatory covariates on SVR12: demographic information: age, gender and BMI; HCV genotype GT1/GT4; treatment duration; HIV/HCV co-infection or HCV mono-infection; liver parameters: albumin, platelet count, GOT/GPT, bilirubin and cirrhosis status (yes/no); renal parameters: creatinine and glomerular filtration; patients on haemodialysis or peritoneal dialysis. No multiple logistic regression was calculated as no covariates were statistically significant.

SAS statistical software, version 9.2 (SAS Institute Inc., Cary, NC, USA) was used for all data analysis.

## Results

### Patient population

A total of 137 patients were included at 31 Hepatology and ID Units across Spain, 135 of whom were evaluable; two patients were excluded for not having started 3D/2D ± RBV treatment in 2015.

Table 1 shows the demographic and clinical characteristics of HCV-infected patients at baseline by CKD stage: the mean (SD) age of patients included in the study was 58.3 (11.4) years, most of them were Caucasian, mean (SD) BMI was 25.4 (4.64) kg/m^2^, with no significant differences across renal stages. Baseline HCV RNA levels were less than 800,000 IU/ml in 81 patients (60%). In total, 92.6% of patients were GT1 and 7.4% were GT4. Fourteen (10.4%) had HIV/HCV co-infection, mainly transmitted through intravenous drug use (76.8%) and sexual transmission (21.4%). At the start of the 3D/2D treatment, 11.1% patients had CKD stage 3b, 14.8% stage 4 and 74.1% stage 5. In total, 93 patients (68.9%) were on haemodialysis and mean time on haemodialysis was 6.9 years. One hundred and twenty-five (125) patients (92.6%) received the 3D regimen and 10 patients (7.4%) received 2D. Out of the 41 patients (30.4%) that received concomitant RBV, 33 patients (24.4%) were receiving the 3D regimen and 8 (5.9%) the 2D regimen. The most commonly prescribed doses of RBV were 200 mg (75.0%) and 400 mg (10.0%) and the median RBV duration was 12 weeks.

**Table 1:** Baseline demographics, HCV characteristics and previous and current treatment characteristics.

Variable	Stage IIIb(^1^) n = 15	Stage IV n = 20	Stage V n = 100	Total n = 135
Age (yrs)				
Mean (SD)	65.3 (10.8)	64.5 (11.6)	56.1 (10.5)	58.3 (11.3)
Gender (%)				
Male	9 (60.0%)	12 (60.0%)	72 (72.0%)	93 (68.9%)
Ethnicity (%)				
Caucasian	15 (100%)	20 (100%)	98 (98.0%)	133(98.5%)
Genotype (%)				
GT1	14 (93.3%)	18 (90.0%)	93 (93.0%)	125 (92.6%)
GT 1a	2 (14.3%)	4 (22.2%)	16 (17.2%)	22 (17.6%)
GT 1b	12 (85.7%)	14 (77.8%)	76 (81.7%)	102 (81.6%)
GT4	1 (6.7%)	2 (10.0%)	7 (7.0%)	10 (7.4%)
Fibrosis stage (%)				
F0-F1 F2 F3 F4	1 (7.7%) 3 (23.1%) 4 (30.8%) 5 (38.5%)	10 (58.8%) 3 (17.6%) 2 (11.8%) 2 (11.8%)	32 (35.2%) 15 (16.5%) 17 (.7%) 27 (29.7%)	43 (35.5%) 21 (17.4%) 23 (19.0%) 34 (28.1%)
VHC viral load (cat3)				
VHC >800,000 IU/mL	5 (33.3%)	13 (65.0%)	63 (63.0%)	81 (%)
HIV co-infection	3 (20.0%)	3 (15.0%)	8 (8.0%)	14 (10.4%)
eGFR				
Mean (SD)	40.35 (11.0)	23.60 (5.1)	--	--
Creatinine				
Mean (SD)	1.68 (0.5)	2.83 (1.2)	6.80 (2.7)	--
Total bilirubin [mg/dl]				
Mean (SD)	0.37 (0.2)	0.44 (0.2)	0.41 (0.2)	0.41 (0.2)
Platelets [x10E3/l]				
Mean (SD)	208733.33 (120427.5)	186350.0 (69421.9)	174737.4 (78643.0)	180276.12 (83017.92)
Hemoglobin [g/dl]				
Mean (SD)	13.01 (1.5)	12.23 (1.9)	11.89 (1.7)	12.1 (1.76)
INR				
Mean (SD)	1.1 (0.19)	1.2 (0.73)	1.1 (0.26)	1.1 (0.36)
Hemodialysis (%) (^2^)	--	1 (5.0%)	92 (92.0%)	93 (68.9%)
Dialysis (%) (^2^)	--	1 (5.0%)	100 (100%)	101 (74.8%)
Peritoneal dialysis	--	--	12 (12.0%)	12 (8.9%)
Renal transplant (%)	--	--	52 (52.0%)	52 (38.5%)
**PREVIOUS STUDY TREATMENT**				
Naïve–not treated	11 (73.3%)	16 (80.0%)	70 (70.0%)	97 (71.9%)
Response:				
Null response	--	--	9 (30.0%)	9 (23.7%)
Partial response	1 (25.0%)	--	10 (33.3%)	11 (28.9%)
Recurrence	1 (25.0%)	1 (25.0%)	5 (16.7%)	7 (18.4%)
**CURRENT STUDY TREATMENT**				
3D Regimen(^3^)				
Total	14 (93.3%)	18 (90.0%)	93 (93.0%)	125 (92.6%)
3D-RBV	10 (66.7%)	14 (70.0%)	68 (68.0%)	92 (68.1%)
3D+RBV	4 (26.7%)	4 (20.0%)	25 (25.0%)	33 (24.4%)
2D Regimen(^4^)				
Total	1 (6.7%)	2 (10.0%)	7 (7.0%)	10 (7.4%)
2D-RBV	--	1 (5.0%)	1 (1.0%)	2 (1.5%)
2D+RBV	1 (6.7%)	1 (5.0%)	6 (6.0%)	8 (5.9%)
With RBV	5 (33.3%)	5 (25.0%)	31 (31.0%)	41 (30.4%)
RBV duration (w)				
Mean (SD)	13.5 (6.11)	11.9 (0.17)	16.8(28.46)	15.8 (24.79)

(^1^): Stage IIIb GFR: 30–44 ml / min / 1.73 m^2^; Stage IV GFR: 15–29 ml / min / 1.73 m^2^_;_ Stage V (renal failure) GFR <15 ml / min / 1.73 m^2^ (including those on HD and PD)

(^2^): A patient in hemodialysis was included as stage IV.

(^3^) 3D: Ombitasvir + Paritaprevir + Dasabuvir

(^4^) 2D: Ombitasvir + Paritaprevir

A high percentage of patients (88.9%) reported previous diseases in the 12 months prior to 3D/2D regimen initiation. The most prevalent diseases were: hypertension 70.4%; diabetes mellitus 24.4%, hyperparathyroidism 17.8% and hyperlipidaemia 14.1%; other endocrine comorbidities (27.4%); renal and urinary disorders (21.5%) and cardiac disorders (19.3%).

Due to the potential impact of drug-drug interactions occurrence, especially in the CKD population, the percentage and class of concomitant medication and its management were evaluated. A total of 127 patients (94.1%) received medication for concomitant diseases during the 2D/3D treatment and follow-up, including new medication and medicines initiated prior to 3D/2D. The most common concomitant medications administrated during the 3D/2D regimen were: beta blockers (38.5%), calcium channel blockers (38.5%), agents acting on the renin-angiotensin system (31.9), diuretics (27.4%), antihypertensives (20.0%) and lipid-lowering agents (19.3%).

Prior to 2D/3D study treatment, 12.7% of concomitant medication was prescribed for prophylaxis and 89.8% continued being prescribed during the 2D/3D study. It is noteworthy that 93.5% of medication administered during the study continued to be prescribed at the end of the study.

### Overall sustained virological response rates (effectiveness)

Plasma HCV RNA was undetectable in 79.2% (99/125) of patients by week 4 and in 98.4% (127/129) at EOT. Out of the 135 patients who initiated the 3D/2D regimen, 125 (92.6%) achieved ITT SVR12 (95% CI: 88.2%-97.0%). Two of the 135 patients achieved SVR12 although treatment was discontinued due to adverse reactions.

Ten patients did not achieve SVR12: 1 patient (0.7%) presented virological failure as a rebound in week 11 post-treatment (47 IU/ml) (a 42-year-old male, stage 5 on haemodialysis, with HCV GT1b infection, who received 3D for 12 weeks); five patients (3.7%) discontinued the treatment (four due to four serious adverse events (SAEs), including three deaths, and one at the patient’s request) and four patients had missing data and/or were lost to follow-up.

**Fig 1:**
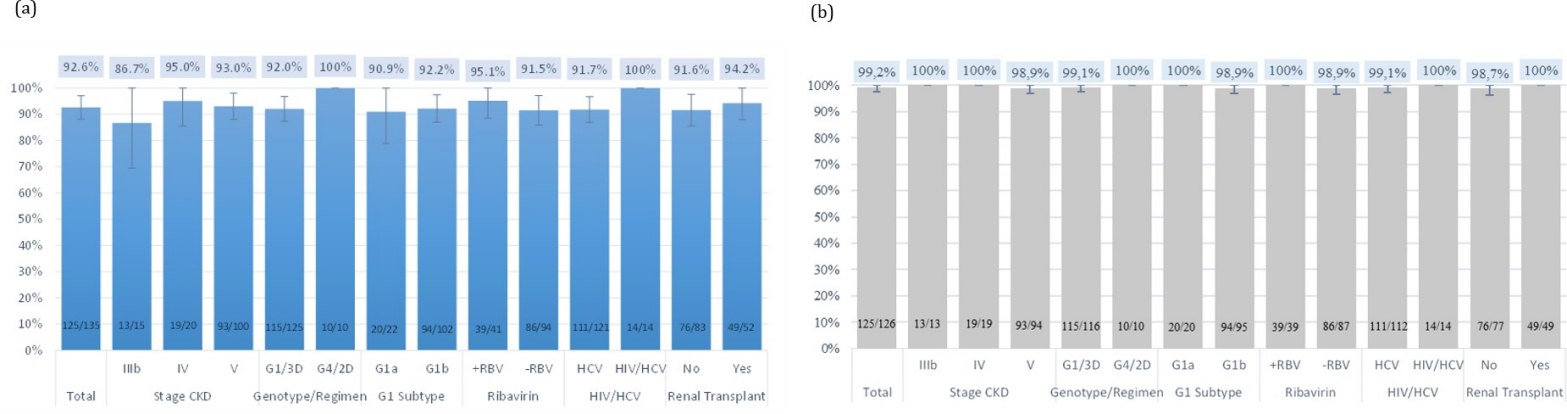
Overall SVR12 for each sub-group of patients studied. (a) SVR12 in the ITT population. The groups analyzed are: CKD stage, Genotype/Regimen, RBV use, coinfection and renal transplant. The overall ITT SVR12 was 92.6% (125/135; 95% CI 88.2–97.0) for patients receiving 3D/2D regimen. (b) SVR12 in the mITT population which includes only those patients in the ITT population excluding those patients whom SVR12 assessment data were missing (unknown) and those patients who discontinued. All percentages are over 98%.

The mITT SVR12 rate was 99.2% (125 of 126; 95% CI 97.7%-100%). Fig 1A and 1B show the overall ITT and mITT SVR12 for each of the sub-groups: no statistically significant differences were found across renal stages, genotypes, G1 subtype, HCV and HIV/HCV coinfection, 3D/2D regimen, RBV treatment or renal transplant.

Table 2 shows ITT SVR12 stratified by renal stage and RBV use: no statistically significant differences were found in the percentage of patients achieving SVR12 by RBV use, within each renal stage. Overall, the SVR12 rate was 91.5% for patients treated with the 3D/2D regimen alone and 95.1% for patients treated with the 3D/2D regimen plus RBV.

**Table 2:** SVR12 by renal stage according the RVB use.

		Stage IIIb	Stage IV	Stage V	Total
+RBV	Total	5 (100%)	5 (100%)	31 (100%)	41 (100%)
	SVR12	5 (100%)	5 (100%)	29 (93.5%)	39 (95.1%)
	Withdrawal	--	--	1 (3.2%)	1 (2.4%)
	Unknown	--	--	1 (3.2%)	1 (2.4%)
-RBV	Total	10 (100%)	15 (100%)	69 (100%)	94 (100%)
	SVR12	8 (80.0%)	14 (93.3%)	64 (92.8%)	86 (91.5%)
	Virological failure	--	--	1 (1.4%)	1 (1.1%)
	Withdrawal	1 (10.0%)	1 (6.7%)	2 (2.9%)	4 (4.3%)
	Unknown	1 (10.0%)	--	2 (2.9%)	3 (3.2%)
Total	Total	15 (100%)	20 (100%)	100 (100%)	135 (100%)
	SVR12	13 (86.7%)	19 (95.0%)	93 (93.0%)	125 (92.6%)
	Virological failure	--	--	1 (1.0%)	1 (0.7%)
	Withdrawal	1 (6.7%)	1 (5.0%)	3 (3.0%)	5 (3.7%)
	Unknown	1 (6.7%)	--	3 (3.0%)	4 (3.0%)
Global information	+RBV	5/5 (100%)	5/5 (100%)	29/31 (93.5%)	39/41 (95.1%)
	-RBV	8/10 (80.0%)	14/15 (93.3%)	64/69 (92.8%)	86/94 (91.5%)
	Total	13/15 (86.7%)	19/20 (95.0%)	93/100 (93.0%)	125/135 (92.6%)
	p-value (for each stage)	1.000	1.000	1.000	--

### eGFR evolution during treatment

**Fig 2:**
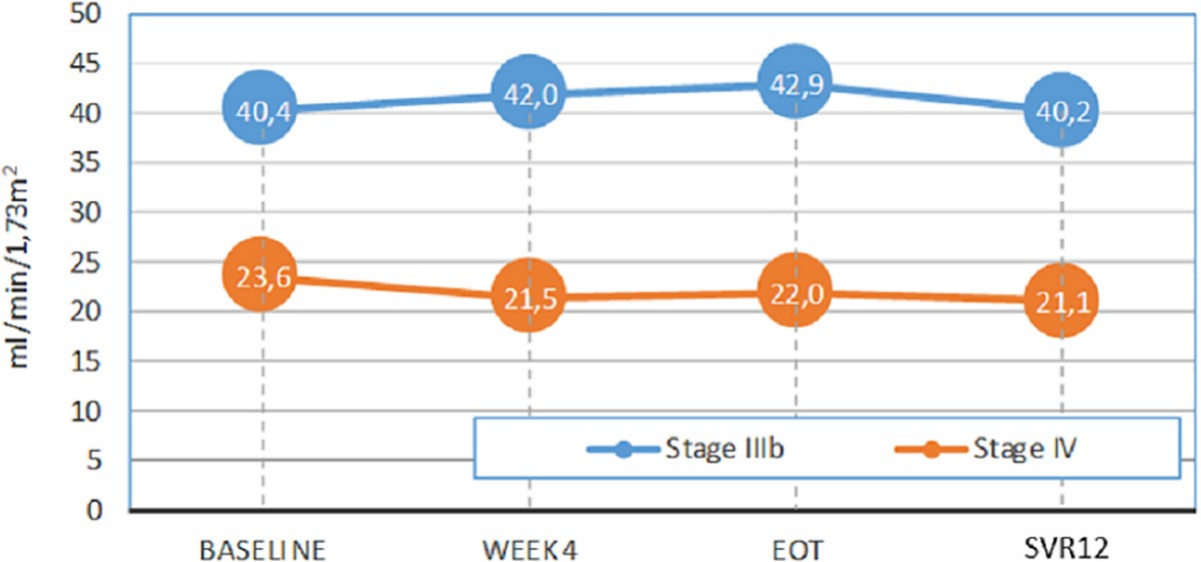
Evolution of eGFR (ml/min/1.73 m^2^). Evolution of mean levels of eGFR in Stage IIIb and Stage IV during period study: from baseline to SVR12.

The eGFR was calculated for all Stage IIIb and IV patients (n = 15 and n = 19 patients, respectively) (excluding one stage-IV patient on dialysis at the physician’s discretion). Mean eGFR for stages IIIb and IV were 40.35 and 23.6 ml/min/1.73m^2^, respectively, at baseline, with a slightly variation during 3D/2D treatment at EOT (42.91 and 21.97 ml/min/1.73m^2^), although these differences were not statistically significant. Mean eGFR values at the SVR12 visit were 40.24 and 21.07 ml/min/1.73m^2^ for Stage IIIb and IV, respectively. (Fig 2).

Table 3 shows changes in eGFR from baseline stratified by renal stages > 10 ml/min/1.73m^2^. Overall, a clinical significant change in eGFR (a decrease > 10 ml/min/1.73m^2^) was reported for only two patients, both in stage IIIb. Therefore, glomerular filtration deterioration was not reported for stage 4.

**Table 3:** Change in eGFR from baseline (>10 ml/min/1.73m^2^) by renal stage.

		Stage IIIb n = 15	Stage IV n = 20
Change eGFR 4W	Decreases > 10 ml/min/1.73m^2^	2 (13.3%)	1 (5.3%)
	Not changing	8 (53.3%)	18 (94.7%)
	Increases > 10 ml/min/1.73m^2^	5 (33.3%)	0 (0%)
Change eGFR EOT	Decreases > 10 ml/min/1.73m^2^	2 (13.3%)	0 (0%)
	Not changing	9 (60.0%)	19 (100%)
	Increases > 10 ml/min/1.73m^2^	4 (26.7%)	0 (0%)
Change eGFR SVR12 visit	Decreases > 10 ml/min/1.73m^2^	2 (13.3%)	0 (0%)
	Not changing	12 (80.0%)	19 (100%)
	Increases > 10 ml/min/1.73m^2^	1 (6.7%)	0 (0%)

Only two patients decreased more than 10 units in eGFR at SVR12 visit, both stage III

### Safety

A total of 64 patients (47.4%) receiving the 3D/2D regimen ± RBV experienced AEs, and 31.9% of all reported AEs were mild. AEs reported by more than 10% of patients were: asthenia (13.3%), anaemia (12.6%) and pruritus (11.1%).

During the treatment period, 14 patients (10.4%) changed the treatment prescribed at baseline: two patients (1.55%) changed 3D/2D regimen and 12 patients (29.3%) changed their initial RBV treatment. Both patients who changed their 3D/2D regimen suspended 3D treatment due to adverse events.

Regarding RBV changes, eight patients reduced the dose, three patients suspended it and one increased the dose. The reasons for these changes were adverse events (seven patients), haemoglobin (Hg) reduction (two patients), increased eGFR and simplification of treatment pattern (one patient).

Six patients (4.4%) discontinued treatment due to AEs: two of the patients with anaemia achieved SVR12. Four patients included in this study died. One patient died due to endocarditis after completion of the 3D/2D treatment and three died during 3D/2D treatment: one patient due to liver failure and acute kidney injury (probably attributable to the 3D/2D regimen by the investigator); one due to atrioventricular block (not related to the 3D/2D regimen by the investigator); and one due to subdural haematoma (not related to the 3D/2D regimen by the investigator).

**Fig 3:**
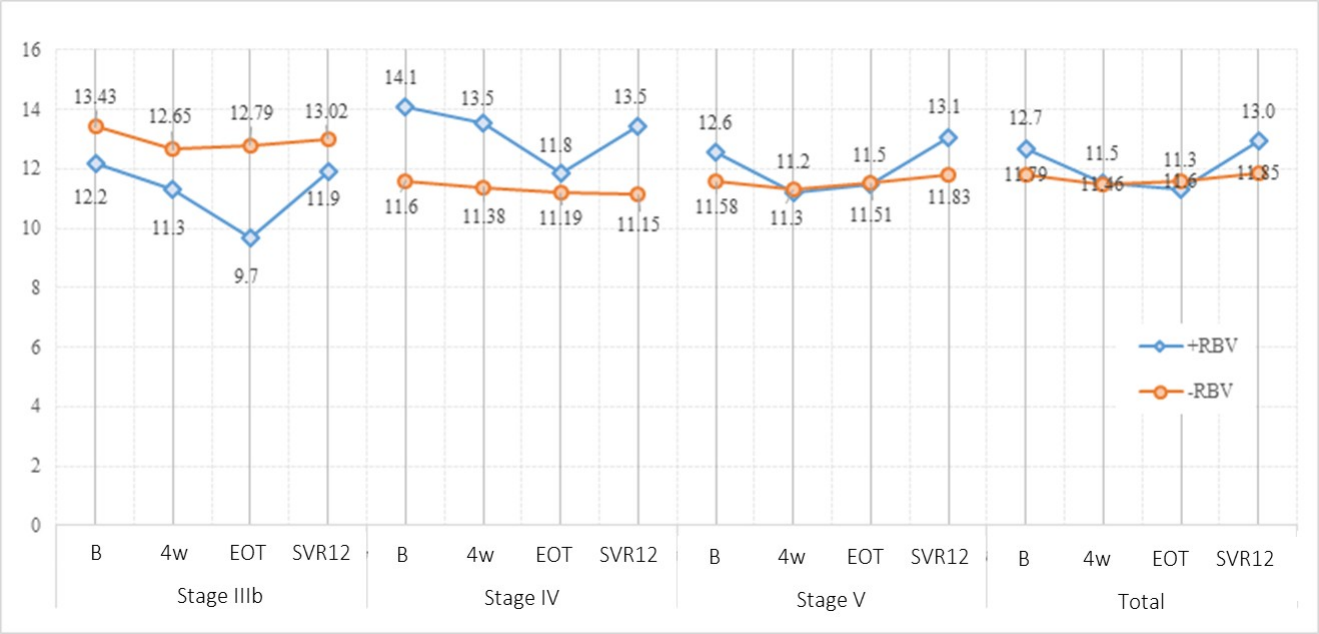
Evolution and change from baseline in hemoglobin (g/dl) by stage and RBV use. In the total population receiving RBV, there was a statistically significant decrease in Hb mean values (11.5 and 11.3 g/dl) in week 4 and EOT (p<0.001), but values recovered at SVR12 visit (13.0 g/dl) baseline ranges (p>0.05). No differences were obtained in patients not treated with RBV along the follow-up period.

Although mean Hb values decreased overall during the treatment period in the population receiving RBV (from 12.7 g/dl at baseline to 11.5 and 11.3 g/dl at week 4 and EOT, respectively; p<0.001), these values had recovered at the SVR12 visit (13 g/dl). Fig 3 shows the evolution and changes from baseline in Hb by stage and by RBV use.

## Discussion

The findings of our real-world study in terms of the efficacy of 3D/2D ± RBV replicated not only the results of the RUBY-I [22] and RUBY-II [24] clinical trials, but also those obtained in a small Spanish cohort in real clinical practice reported by Muñoz et al.[27]. The aforementioned study included 46 patients, 10 of whom (21.7%) with CKD stage IV and 36 (78.2%) with CKD stage V and found an SVR12 rate of 95.7% in the ITT population. In our study of 135 patients with severe renal failure or end-stage renal disease infected by HCV GT 1 or 4 treated with 3D/2D ± RBV, the overall SVR12 was 92.9% (ITT, 125/135) and effectiveness was found to be similar in all analysed sub-groups, with only one virological failure. These findings suggest that 3D/2D ± RBV treatment is highly effective and feasible in clinical practice, with mild adverse events in our studied population.

No statistically significant difference was observed in SVR12 in either 3D/2D regimens when RBV was added to treatment (95.1% and 91.5%, respectively) in our study. These data suggest that RBV may not be necessary in some GT1a- or GT4-infected patients with severe renal impairment treated with a 3D/2D regimen, consistent with the conclusions from the RUBY-II study [24].

It is known that HIV/HCV co-infection is associated with higher liver-related morbidity and mortality, making it very important to treat HCV infection. Current DAAs for HCV treatment have shown similar SVR results in HIV/HCV co-infected patients in various studies [28,29]. In Germany, results from a recent study showed that SVR12 rates did not differ between HCV mono-infected and HIV/HCV co-infected patients with liver cirrhosis (87.8% vs 89.3%, respectively; P = 0.864) [30]. In another study, HIV/HCV co-infected patients with/without cirrhosis treated with grazoprevir and elbasvir achieved an SVR12 of 96%, showing that the HCV treatment regimen seems to be effective and well tolerated for patients co-infected with HIV with or without cirrhosis [31]. On the basis of these results, the international guidelines recommend the same treatment regimens for both HIV/HCV co-infected patients and patients with HCV alone [32]. Our study supports this recommendation, as the 14 HIV/HCV co-infected patients all achieved SVR12 (100%).

Glomerular filtration was calculated in non-dialysed patients and, despite a statistically significant decrease in mean eGFR by week 4 in stage IV patients, the mean eGFR during treatment remained clinically stable. In fact, only two patients in stage IIIb showed a clinically significant change in eGFR (a decrease > 10 ml/min/1.73m^2^). Real-world evidence provided by the study by Welzel TM et al. in GT1- or GT4-infected patients with renal insufficiency support our findings, as no clinically relevant changes in renal function, defined by a decrease in eGFR of more than 10 units between baseline and end of therapy, were observed, supporting the clinical use of the 3D/2D combination in our studied population [33].

The adverse events profile was similar to that described in other studies, where anaemia, asthenia and pruritus were the most common AEs during 3D/2D treatment, similar to that reported by Pockros et al.[22] Two patients discontinued the 3D/2D regimen due to anaemia. Four patients died during the study period: three of them not related to the 3D/2D regimen and one probably related to treatment. Treatment discontinuation was low (<10% in the overall population) and regimens that included ribavirin had more mild or moderate adverse events than those without. The safety and tolerability of the 3D or 2D regimen in real-world conditions are similar to that obtained under clinical trial conditions. Furthermore, the RBV-free 3D/2D regimens were generally well tolerated in this patient population, which is similar to the findings of other studies. The majority of patients (93.5%) did not discontinue their concomitant medication during this real-world study, suggesting that drug–drug interactions in this population were manageable.

Due to the importance of cardiovascular comorbidities in this HCV population, particularly in CKD patients, haematological toxicity is strictly controlled [10]. Although haemoglobin values decreased significantly during the treatment period, values recovered at 12 weeks post-treatment, irrespective of RBV treatment, and did not affect the SVR. By contrast, platelet levels increased significantly from baseline to week 4, recovering the baseline values at 12 weeks post-treatment.

This study has several limitations, due to its retrospective nature, which may have introduced selection bias, for example a mostly Caucasian race representative of Spanish population, and issues regarding missing data, as in the case of the management of concomitant medication or the collection of all potential treatment related AEs. Additionally, patients were treated at each physician’s discretion, so dose adjustments and the management of AEs were not pre-specified by a protocol. It should also be mentioned that although the study's sample size is limited to 135 patients, the 100-dialysis patient-sample size is noteworthy.

In this real-world study, high rates of SVR12 and good safety profiles were achieved. These results are comparable not only with those obtained in clinical trials [21–23], but also with studies conducted in real-world conditions [34,35]. However, it is striking that our population included different subgroups of HCV patients, most of them on dialysis, showing that these regimens are effective in our diverse population, providing important guidance for clinical decision-making. These data are relevant for those countries in which 3D/2D is currently a heavily prescribed regimen for the treatment of HCV infection in CKD patients.

## Conclusion

This study demonstrated that 3D/2D regimens with or without RBV are effective and safe in real-world conditions in chronic HCV genotype 1 or 4 mono-infected and HIV/HCV co-infected patients with stage 3b, 4 and 5 (end-stage) chronic kidney disease, including on dialysis. The high ITT SVR12 rate obtained (92.6%) is particularly noteworthy, which is consistent with data obtained under clinical trial conditions. 3D/2D regimens were also well tolerated in these patients, who were treated in routine clinical practice at various Spanish centres, with most AEs being mild or moderate.

## Supporting information

S1 Text List of institutional ethic committees. (DOCX)
