# Supplementary material for: Effectiveness, safety/tolerability of OBV/PTV/r ± DSV in patients with HCV genotype 1 or 4 with/without HIV-1 co-infection, chronic kidney disease (CKD) stage IIIb-V and dialysis in Spanish clinical practice – Vie-KinD study
Source: PLoS One. 2019 Sep 24;14(9):e0221567. doi: 10.1371/journal.pone.0221567 (PMC6759177; doi:10.1371/journal.pone.0221567)
Supplement: S1 Text — (DOCX) [file pone.0221567.s001.docx]

S1 Supporting information – List of Institutional ethic committees

1. Comité de Ética de la Investigación (CEI) del Principado de Asturias, Asturias, Spain.
2. Comité de Ética de investigación Clínica del Consorci Sanitari de Terrassa, Barcelona, Spain.
3. Comité Ético de Investigación Clínica del Hospital Clínic de Barcelona, Barcelona, Spain.
4. Comité Ético de Investigación Clínica del Hospital Universitari de Bellvitge, Barcelona, Spain.
5. Comité Ético de Investigación Clínica - Vall d’Hebron Institut de Recerca, Barcelona, Spain.
6. Comité de Ética de la Investigación de Córdoba, Córdoba, Spain.
7. Comité Ético de Investigación del Hospital de la Princesa, Madrid, Spain.
8. Comité de Ética de la Investigación Clínica de la Fundación Jiménez Díaz, Madrid, Spain.
9. Comité Ético de Investigación Clínica - Hospital Clínico San Carlos de Madrid, Madrid, Spain.
10. Comité Ético de Investigación Clínica - Hospital General Universitario Gregorio Marañón, Madrid, Spain
11. Comité Ético de Investigación Clínica - Hospital Universitario 12 de Octubre, Madrid, Spain.
12. Comité ético de Investigación Clínica del Hospital Universitario La Paz, Madrid, Spain.
13. Comité Ético de Investigación de los Hospitales Virgen Macarena y Virgen del Rocío, Seville, Spain.
14. Comité Ético de Investigación Clínica del Hospital Universitario Sta. Mª del Rosell, Áreas II y VIII de Salud del Servicio Murciano de Salud, Murcia, Spain.
15. Comité Ético de Investigación Clínica del Hospital Universitario y Politécnico La Fe, Valencia, Spain.
